# Systematic Review of Magnetic Resonance Lymphangiography From a Technical Perspective

**DOI:** 10.1002/jmri.27542

**Published:** 2021-02-24

**Authors:** Michael Mills, Malou van Zanten, Marco Borri, Peter S. Mortimer, Kristiana Gordon, Pia Ostergaard, Franklyn A. Howe

**Affiliations:** 1 Molecular and Clinical Sciences Research Institute, St George’s University, London, UK; 2 Department of Neuroradiology, King’s College Hospital, London, UK; 3 Lymphovascular Medicine, Dermatology Department, St George’s Hospital, London, UK

## Abstract

**Background:**

Clinical examination and lymphoscintigraphy are the current standard for investigating lymphatic function. Magnetic resonance imaging (MRI) facilitates three-dimensional (3D), nonionizing imaging of the lymphatic vasculature, including functional assessments of lymphatic flow, and may improve diagnosis and treatment planning in disease states such as lymphedema.

**Purpose:**

To summarize the role of MRI as a noninvasive technique to assess lymphatic drainage and highlight areas in need of further study.

**Study Type:**

Systematic review.

**Population:**

In October 2019, a systematic literature search (PubMed) was performed to identify articles on magnetic resonance lymphangiography (MRL).

**Field Strength/Sequence:**

No field strength or sequence restrictions.

**Assessment:**

Article quality assessment was conducted using a bespoke protocol, designed with heavy reliance on the National Institutes of Health quality assessment tool for case series studies and Downs and Blacks quality checklist for health care intervention studies.

**Statistical Tests:**

The results of the original research articles are summarized.

**Results:**

From 612 identified articles, 43 articles were included and their protocols and results summarized. Field strength was 1.5 or 3.0 T in all studies, with 25/43 (58%) employing 3.0 T imaging. Most commonly, imaging of the peripheries, upper and lower limbs including the pelvis (32/43, 74%), and the trunk (10/43, 23%) is performed, including two studies covering both regions. Imaging protocols were heterogenous; however, T_2_-weighted and contrast-enhanced T_1_-weighted images are routinely acquired and demonstrate the lymphatic vasculature. Edema, vessel, quantity and morphology, and contrast uptake characteristics are commonly reported indicators of lymphatic dysfunction.

**Data Conclusion:**

MRL is uniquely placed to yield large field of view, qualitative and quantitative, 3D imaging of the lymphatic vasculature. Despite study heterogeneity, consensus is emerging regarding MRL protocol design. MRL has the potential to dramatically improve understanding of the lymphatics and detect disease, but further optimization, and research into the influence of study protocol differences, is required before this is fully realized.

**Level of Evidence:**

2

**Technical Efficacy:**

Stage 2

## Introduction

Lymphedema is a condition characterized by the accumulation of lymph in the tissue leading to chronic swelling.^1^ As of 2012, lymphedema was estimated to affect as many as 250 million people worldwide, the majority of which are caused by filariasis in developing nations.^2^ Lymphedema is also prevalent in developed nations; as many as one in 1000 Americans may be affected.^1^ Despite the prevalence, methods of investigating human lymphedema are few and comparatively small numbers of medical professionals specialize in disorders of the lymphatic system.^3^ In vivo imaging of the lymphatics may improve understanding of the underlying causes and mechanisms of lymphatic disorders, and aid diagnosis.

**Figure 1:**
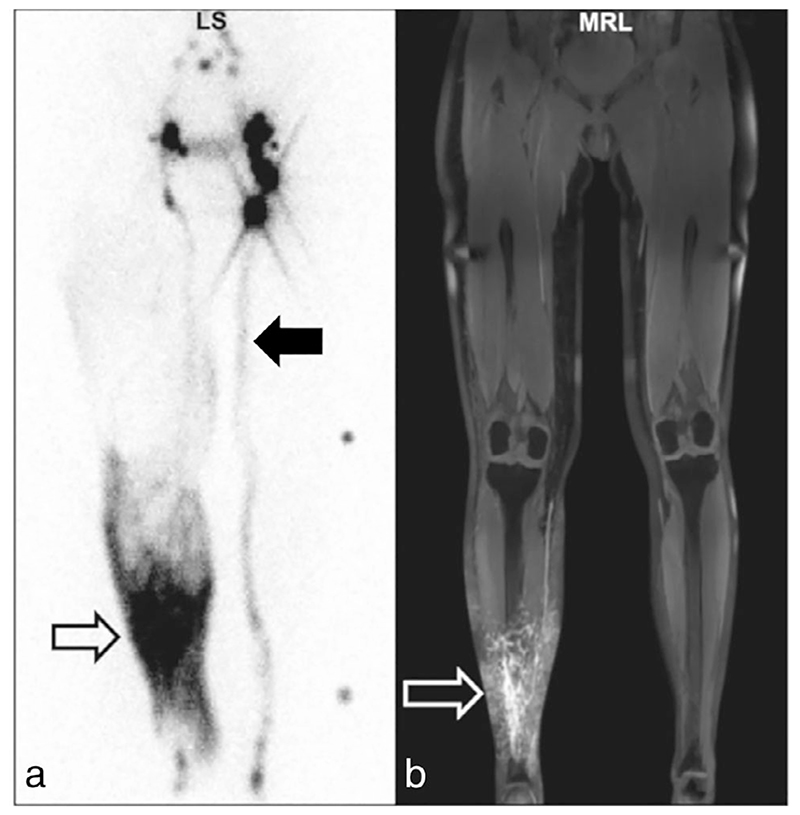
Lymphoscintigram (a) and magnetic resonance lymphangiogram (b) acquired in the lower limbs of a participant with lymphedema of the right lower limb. MRI was acquired after contrast injection in the affected limb with a contrast-enhanced 3D T_1_-weighted gradient echo sequence with TR/TE = 4.13/1.47 msec, flip angle = 25°, reconstructed voxel size = 0.80 × 0.80 × 0.80 mm. Both modalities show regions of dermal reflex (open arrows). The lymphoscintigram also shows a normal appearing main lymphatic pathway leading to the inguinal lymph nodes in the unaffected (left) limb (filled arrow). Reproduced from Weiss *et al*.,^5^ with permission.

Lymphoscintigraphy (LS) is currently considered the clinical standard for lymphatic imaging, with direct X-ray lymphography typically phased out given its invasive nature.^4^ While the radiolabeled bolus used in LS is selectively taken up by the lymphatics, making it highly specific, LS is limited by poor spatial and temporal resolution, and is typically limited to generating two-dimensional (2D) projections of the main lymphatic pathways (see Fig. 1).^5,6^ There is also a small radiation dose associated with LS.

**Figure 2:**
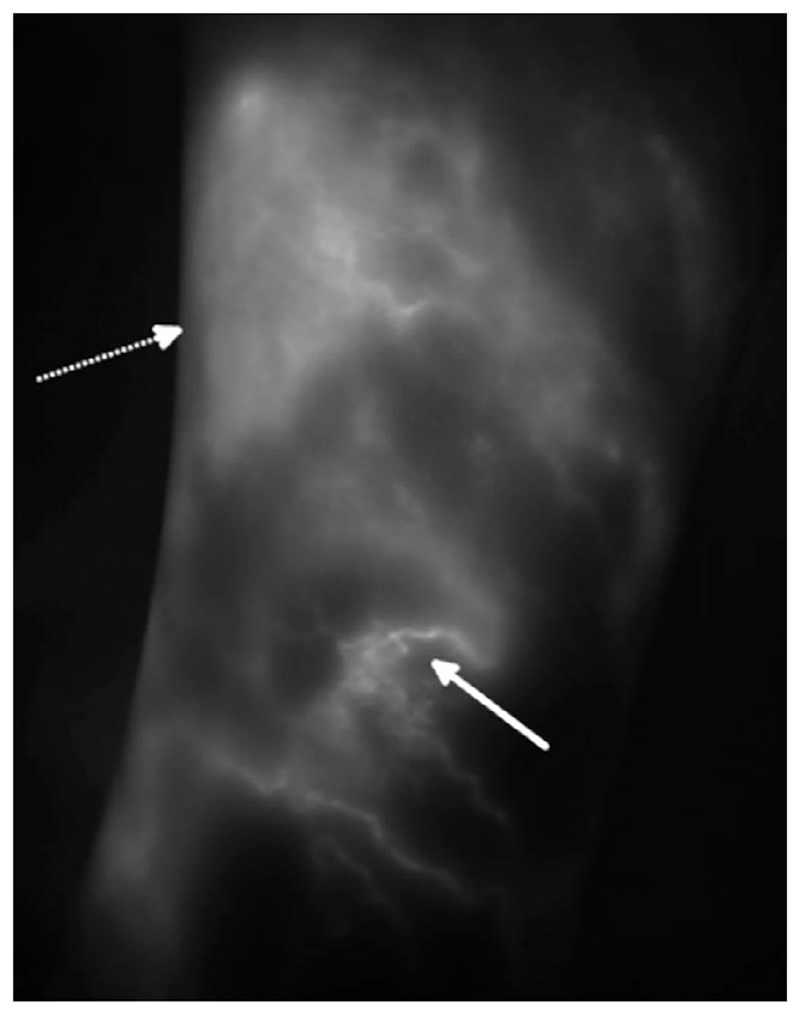
Lower limb indocyanine green (ICG) fluorescence image, showing the lateral aspect of the shin, in a participant with unilateral lower limb lymphedema acquired by St George’s Lymphovascular Research Group. ICG binds to proteins such as albumin making imaging specific to the lymphatics. This image was produced via laser excitation of the ICG after intradermal injection between the digital webspaces, and subsequent detection of the fluorescence by a CCD detector. High spatial resolution allows identification of individual superficial lymphatic vessel (solid arrow); however, emissions from deeper lying structures are quickly attenuated. In the unaffected individual, fairly linear vessel pathways flowing distally to proximally, and following known anatomical pathways, should be observed. In an affected state, an abnormal drainage pattern is evident such as no flow, medial to lateral (or vice versa) flow, and dermal rerouting (dashed arrow). Image “Lower limb ICG in unilateral lymphedema” shared by St George’s Lymphovascular Research Group under the CC BY-SA-4.0 International license (https://creativecommons.org/licenses/by-sa/4.0/). https://commons.wikimedia.org/wiki/File:Lower_Limb_ICG_in_unilateral_lymphoedema.tif.

Indocyanine green (ICG) lymphography, a fluorescence imaging technique (see Fig. 2), overcomes the poor spatial and temporal resolution of LS and is also highly specific to the lymphatics given ICG’s protein binding properties. ICG lymphography is limited by an inability to produce three-dimensional (3D) images and to imaging only superficial lymphatic vessels (LVs).^7^

Magnetic resonance lymphangiography (MRL) is uniquely positioned to yield nonionizing, high spatial resolution 3D imaging of the lymphatic vasculature from head to foot, and appears capable of yielding functional characteristics of lymphatic transport.^8,9^ MRL has garnered increased interest and many small cohort studies, in participants with confirmed or suspected lymphatic abnormalities, have been published.^10–15^ Studies investigating the technical aspects of MRL, and the complexities associated with imaging specific anatomical sites, are less common.

Optimized MRL protocols, with specific study aims, are key to unlocking MRL’s potential for investigating lymphatic function. The aim of this review is to focus on the technical aspects of MRL, discussing potential pitfalls, innovative approaches, and areas in need of further research, while also highlighting any emerging consensus regarding best practice and clinical utility.

## Methods

### Search Strategy

A search of MRL literature was performed using PubMed with search terms: ([lymphography OR lymphangiography] OR Lymphatic angiography OR Lymph angiography) AND (MRI OR MRL OR MR-L OR Magnetic resonance*). English language publications, published between October 7, 1999 and October 7, 2019, were included. Studies published prior to 1999 were not considered. Animal studies were not initially excluded to avoid removing articles which study both human and animal subjects. Manual literature searching provided several additional references.

### Inclusion Criteria

After inspection for duplicates, vetting following the Preferred Reporting Items for Systematic Reviews and Meta-Analyses guidelines was performed.^16^ A single reviewer (MM) performed an initial three-stage filtering:

1. Abstracts not referencing lymphatic magnetic resonance imaging (MRI) or only lymph nodes (LNs) were excluded. Single case reports, letters or replies, and book chapters were also removed.
2. Full texts were retrieved and vetted with the criteria above and the requirement that scanning parameters were present.
3. Studies involving only animals were excluded, retaining those with both human and animal subjects.

### Quality Appraisal

**Figure 3:**
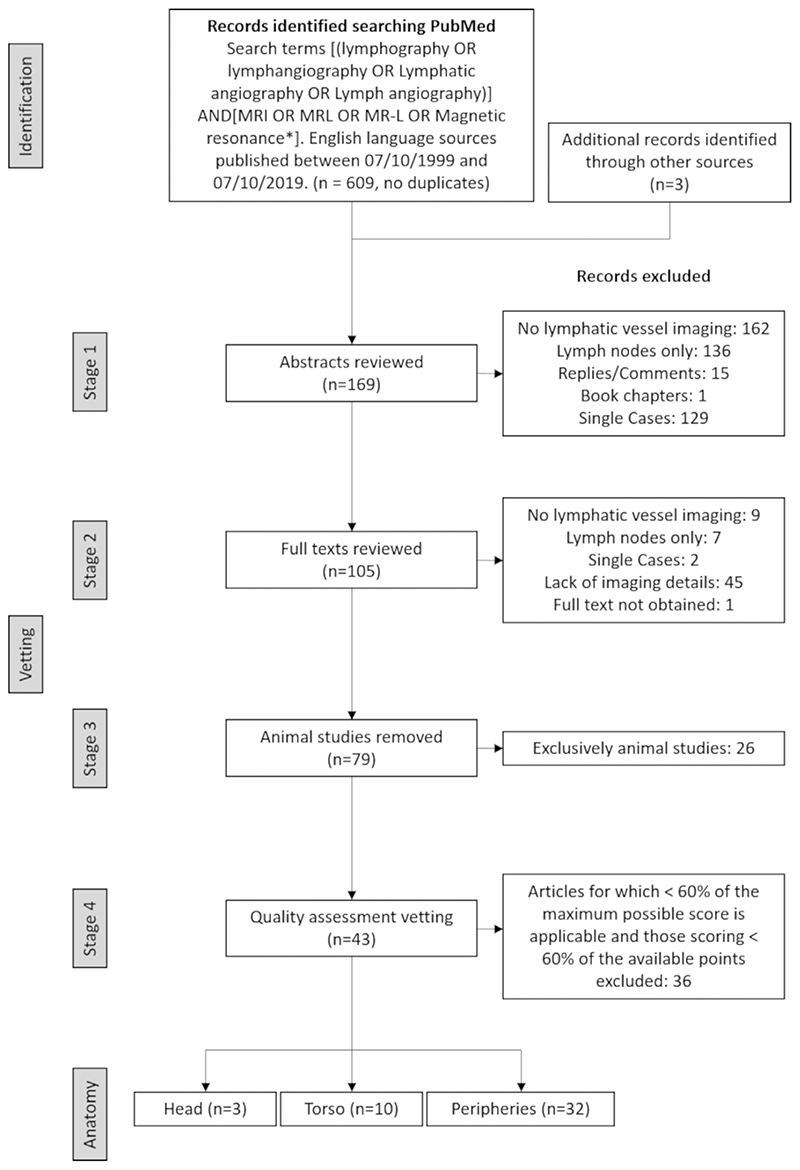
Study selection flow chart. PubMed revealed 609 English language sources after a search for lymphatic vessel magnetic resonance imaging. After vetting and quality assessment, a total of 43 articles were included in this review, the majority of which report imaging in the limbs and/or pelvis (collectively labeled the “peripheries”). Note that some studies cover both the torso and the limbs and so are counted twice. One study, performing peripheral MRL and a single case of torso MRL, was included for review with the single torso case excluded.

Two reviewers (MM and MvZ), with 6 and 3 years of MRI experience, respectively, assessed the quality of the remaining studies using a purpose designed tool produced with heavy reliance on the National Institutes of Health quality assessment tool for case series studies and Downs and Blacks quality checklist for health care intervention studies.^17,18^ Consisting of nine questions, it assessed the clarity of the imaging and contrast injection protocols, potential bias in participant selection, participant compliance, and technical imaging concerns. Articles in this study are those considered of high quality, scoring ≥60% of the points available. Where reviewers disagreed, inclusion was by consensus, or else by a third reviewer (MB) with over 15 years of MRI experience. The full quality appraisal protocol can be found in the Supplementary Material. The article inclusion process is shown in Fig. 3.

## Results

### Included Articles

A total of 43 articles, of an initial 612, were selected after screening and quality appraisal. Magnetic field strength was 1.5 T or 3.0 T in all studies, with 25/43 (58%) studies employing 3.0 T imaging. No human LV studies performed at 7.0 T were identified within any of the initial 612 articles. The most commonly imaged anatomical regions were the peripheries, upper and lower limbs including the pelvis (32/43, 74%), and the trunk (10/43, 23%). Additionally, three studies were performed in the head and neck.

For all included studies, imaging and contrast injection protocols, and summary study findings, have been collated (Tables 1–4). Imaging details for noncontrast and contrast-enhanced (CE) studies can be found in Tables 1–2, while Table 3 outlines contrast injection protocols. These comprehensive tables have been compiled to allow direct comparison of individual studies and show the breadth of applied methodologies.

Reported MRL protocols vary widely; however, 3D heavily T_2_-weighted and CE T_1_-weighted sequences are commonly employed. Maximum intensity projection (MIP) reformatting of the entire imaged volume, including MIP images from each phase of dynamic CE-MRL studies, is regularly employed to aid visualization of the enhancing structures.

## Noncontrast T_2_-Weighted Imaging

**Figure 4:**
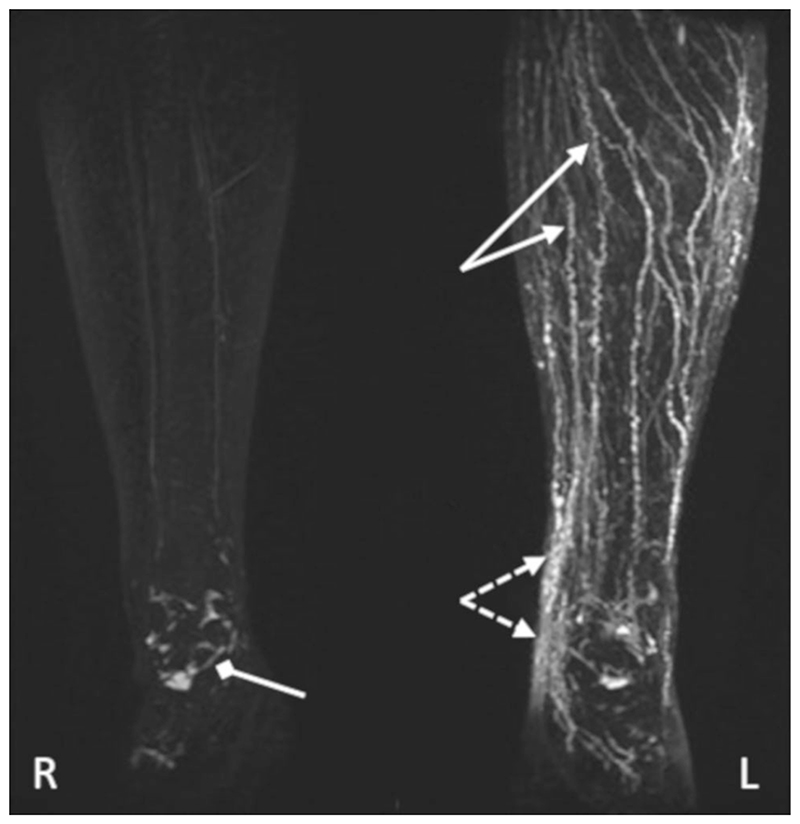
Maximum intensity projected T_2_-weighted noncontrast MRL image of a participant with unilateral lymphedema of the left leg. TR/TE = 4000/884 msec, flip angle = 90°, voxel size = 0.8 × 1.4 mm, acquired with a driven equilibrium pulse. Many tortuous vessel-like structures are seen in the left leg (solid arrows), with signal intense areas of fluid accumulation seen by the left ankle (dashed arrows). High signal structures are also observed at the right ankle (diamond headed arrow). The high signal in the vessel-like structures seen in the left limb may be due to vessel dilation and/or fluid stasis, both of which can occur as a result of pathology. Reproduced from Arrive *et al*.,^28^ with permission.

A fluid-sensitive heavily T_2_-weighted fast/turbo spin echo (FSE/TSE) sequence (note that the generic term “rapid acquisition with relaxation enhancement,” or RARE, is also in use), similar to those used to image the biliary system, is performed in the vast majority of studies acquiring non-CE images (22/29, see Table 1). Ex vivo, the T2 time of lymph has been measured at 610 msec (3.0 T) and hence can be expected to retain reasonable signal in heavily T_2_-weighted images.^9^ An example T_2_-weighted MRL image of the lower limbs clearly displaying LVs can be seen in Fig. 4. Typical timing parameters for these sequences are of the order repetition/echo time (TR/TE) = 3000–4000/500–700 msec at both 1.5 T and 3.0 T with voxel sizes typically >1 mm^3^ (Table 1). Image acceleration techniques such as partial Fourier acquisitions and use of parallel imaging were reported in eight studies employing T_2_-weighted spin echo sequences (11 studies in total, as shown in Table 1); however, the effect on acquisition time is unclear as this was rarely reported. In those which do, 2–11 minute acquisitions have been reported (see Table 1).

Individual studies employed arterial spin labeling (ASL), time of flight (TOF) and steady-state free precession techniques to achieve specific goals such as detecting lymphatic flow in the meningeal lymphatics, estimating the speed of lymphatic flow and to acquire venographic images.

## CE *T_1_-Weighted Imaging*

**Figure 5:**
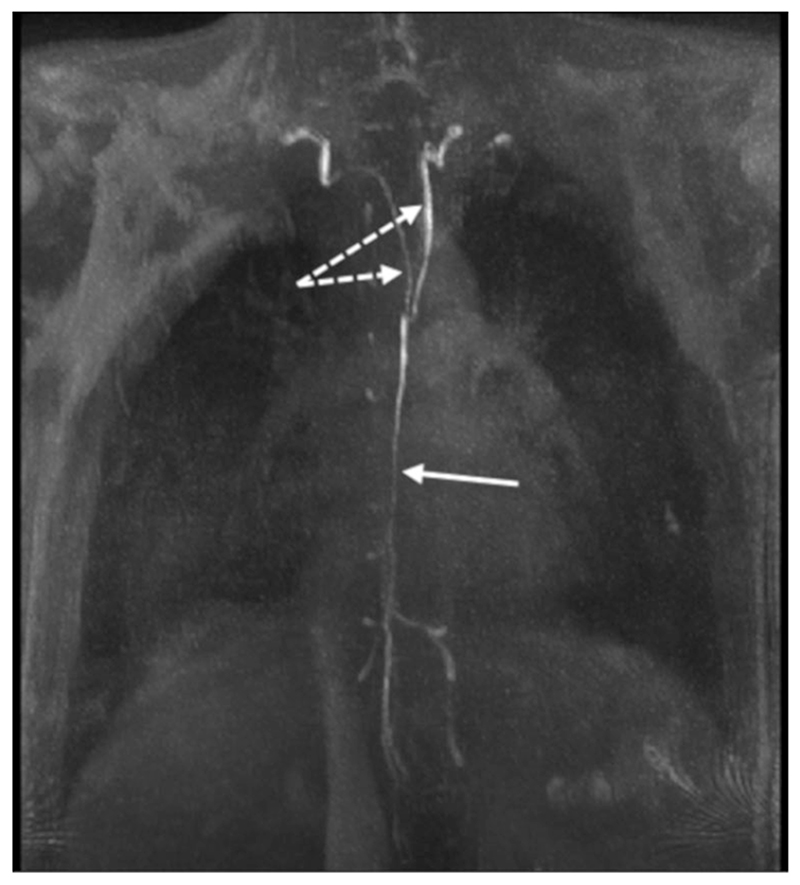
Thoracic duct MRL of a participant with bilateral upper and lower limb lymphedema acquired with a contrast-enhanced T_1_-weighted SPGR by St George’s Lymphovascular Research Group. TR/TE = 5.2 / 1.8 msec, flip angle = 30°, reconstructed voxel size = 0.75 × 0.75 × 1.50 mm. This MIP clearly displays contrast draining through a single smooth channeled thoracic duct (solid arrow), which appears to bifurcate and drain bilaterally (dashed arrows). Image “Thoracic duct MRL in lymphedema” shared by St George’s Lymphovascular Research Group under the CC BY-SA-4.0 International license (https://creativecommons.org/licenses/by-sa/4.0/). https://commons.wikimedia.org/wiki/File:Thoracic_duct_MRL_in_lymphoedema.tif.

Paramagnetic gadolinium-based contrast agents (GBCAs) have been shown capable of reducing the long native T_1_ time of lymph sufficiently to produce high signal intensity T_1_-weighted images, as demonstrated for the thoracic duct (TD) in Fig. 5. Dynamic imaging, demonstrating temporal changes in contrast distribution, is common, with volumes acquired in 30-180 seconds (Table 2).

Short TR and TE 3D spoiled gradient echo (SPGR) sequences, with typical scanning parameters of TR/TE = 3–6 msec/1–2 msec and flip angle (FA) =12–30° regardless of field strength, were most often employed (24/33). Image acceleration techniques were rarely reported in these studies; only one study employing SPGR indicated the use of partial Fourier, while four studies employed parallel imaging. Signal-to-noise ratio (SNR) in CE T_1_-weighted images appears superior to T_2_-weighted ones: Crescenzi *et al*. reported SNR of approximately 10 in the arm and torso LVs using T_2_-weighted TSE at 3.0 T,^25^ while Mazzei *et al*. measured peak SNR in leg LVs of >250 with a CE SPGR sequence at 1.5 T.^27^ Spatial resolution is also typically superior in T_1_-weighted images compared to T_2_-weighted with voxel sizes ~1 mm^3^ reported regularly.

Dixon-based imaging is performed by some authors as a proactive fat suppression technique and by others employing the use of a contrast agent to suppress signal from blood vessels.

## Contrast Injection Protocol

Six different GBCAs were employed within the included studies (Table 3). These agents were often combined with local anesthetic for pain relief, and in one case a small volume of a vasoconstrictor to test if this reduced undesirable venous enhancement^44^; a common issue for peripheral CE-MRL. Gadopentetate dimeglumine and gadobenate dimeglumine were the most often employed GBCAs (12 and 10 instances, respectively), while the use of gadoterate meglumine was only described in a single study.

When performing CE studies in the peripheries, between 2 and 5 injections of ~1 mL GBCA solution were delivered into the digital web spaces, either intradermally or subcutaneously, with small gauge (eg, 24 G) needles. In the trunk (i.e. from pelvis to neck) larger volume injections (eg, 2-8 mL) administered via the inguinal LNs were more common.

Massage of the contrast injection site was performed in approximately half of peripheral MRL (pMRL) studies, often citing research demonstrating improved contrast uptake into the lymphatics of rabbits.^58^ Massage durations varied between 0.5 and 5 minutes.

## Clinical Value

Visualization of LVs is common in T_1_-weighted studies, even in healthy limbs,^34,41,42^ as is recording their abundance and size.

**Figure 6:**
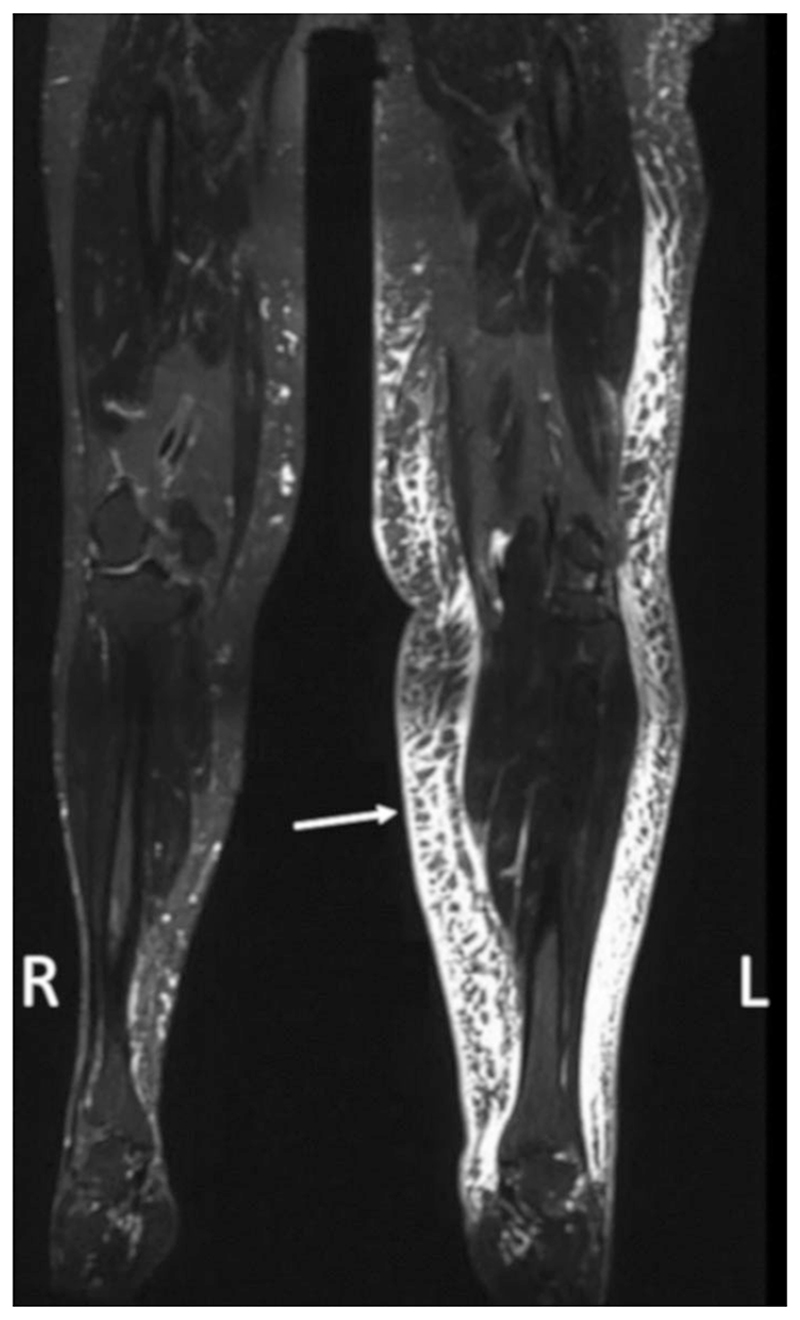
T_2_-weighted TSE image of a participant with lower limb lymphedema in the left limb demonstrating a clear honeycomb pattern of the subcutaneous tissue (arrow). Acquired with TR/TE = 2870/797 msec, voxel size = 1.1 × 1.0 × 1.0 mm. Reproduced from Cellina et *al*.,^59^ with permission.

T_2_-weighted studies appear particularly sensitive to the detection of areas of fluid accumulation and the presence of the so called honeycombing pattern (Fig. 6), thought to be a marker of tissue fibrosis.^60^ LVs are also visualized in T_2_-weighted images; however, this may be improved by specific image optimization: Crescenzi *et al*. acquired images at 3.0 T with a range of echo times and were only able to clearly visualize LVs at TE = 121 msec, an echo time much shorter than is typical.^61^

**Figure 7:**
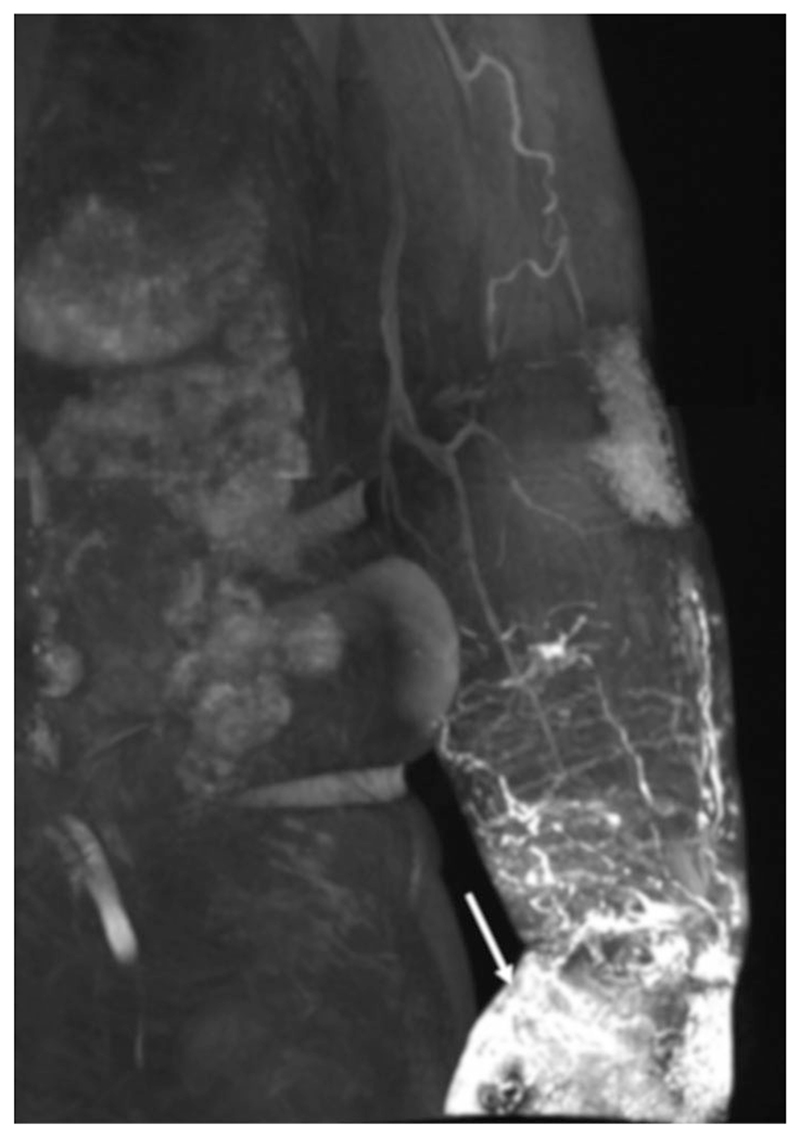
Contrast-enhanced image of the left arm of an individual with lymphedema showing a region of dermal backflow, the rerouting of lymph to the dermal lymphatics. Acquired with a fat suppressed SPGR, TR/TE = 3.5/1.3 msec, flip angle = 14.9°, voxel size = 1.0 × 1.4 × 1.2 mm. Reproduced from Bae *et al*.,^34^ with permission.

LVs are often reported as being larger in participants with lymphedema, and regions of dermal backflow (rerouting of lymphatic fluid to the dermal lymphatics) are regularly observed (Fig. 7).

Dynamic CE studies regularly document the temporal nature of lymphatic enhancement (eg, time to peak signal, or signal vs. time curves), with two authors reporting lymph flow speed estimates: Liu *et al*. estimated speeds between 0.3 and 1.48 cm/minute in the legs of primary lymphedema participants, while Borri *et al*. recorded a speed of 9.7 cm/minute in the arm of a single participant with breast cancer related lymphedema (BCRL).^8,35^ Rane *et al*. measured lymph speed in the arms of healthy controls and BCRL patients using pulsed ASL. Altered lymph dynamics were demonstrated, with a reduction in lymph speed observed in the affected vs. unaffected arms of patients; mean = 0.61 ± 0.22 cm/minute vs. 0.48 ± 0.15 cm/minute.^9^

Kuo *et al*. were able to demonstrate lymph flow in the head, adjacent to the superior sagittal sinus (SSS), via TOF imaging.^19^ Employing spatially selective saturation bands, the direction of flow within the meningeal lymphatics was also demonstrated as being counter to the blood flow of the SSS.

Table 4 summarizes common findings in the included studies.

## Comparisons to Lymphoscintigraphy

Several studies include comparisons of the performance of MRL with LS and comment on the concordance between imaging findings across modalities. In all studies, improved LV visualization with MRL in the limbs was reported.^5,6,34,43^ Improved detection of inguinal LNs was reported by Liu *et al*. when comparing CE-MRL to LS (16/17 vs. 9/17 patient images displaying inguinal nodes for CE-MRL vs. LS), while Notohamiprodjo *et al*. report the converse.^6,43^ In a study considering LS as the gold standard technique, Weiss *et al*. reported sensitivity and specificity values of 68% and 91% for detection of focal lymphatic lesions (eg, lymphocele or dermal backflow) by CE-MRL compared to LS.^5^ Figure 1 shows example LS and MRL images from the same participant.

## Site Specific Considerations

### Peripheral MRL

**Figure 8:**
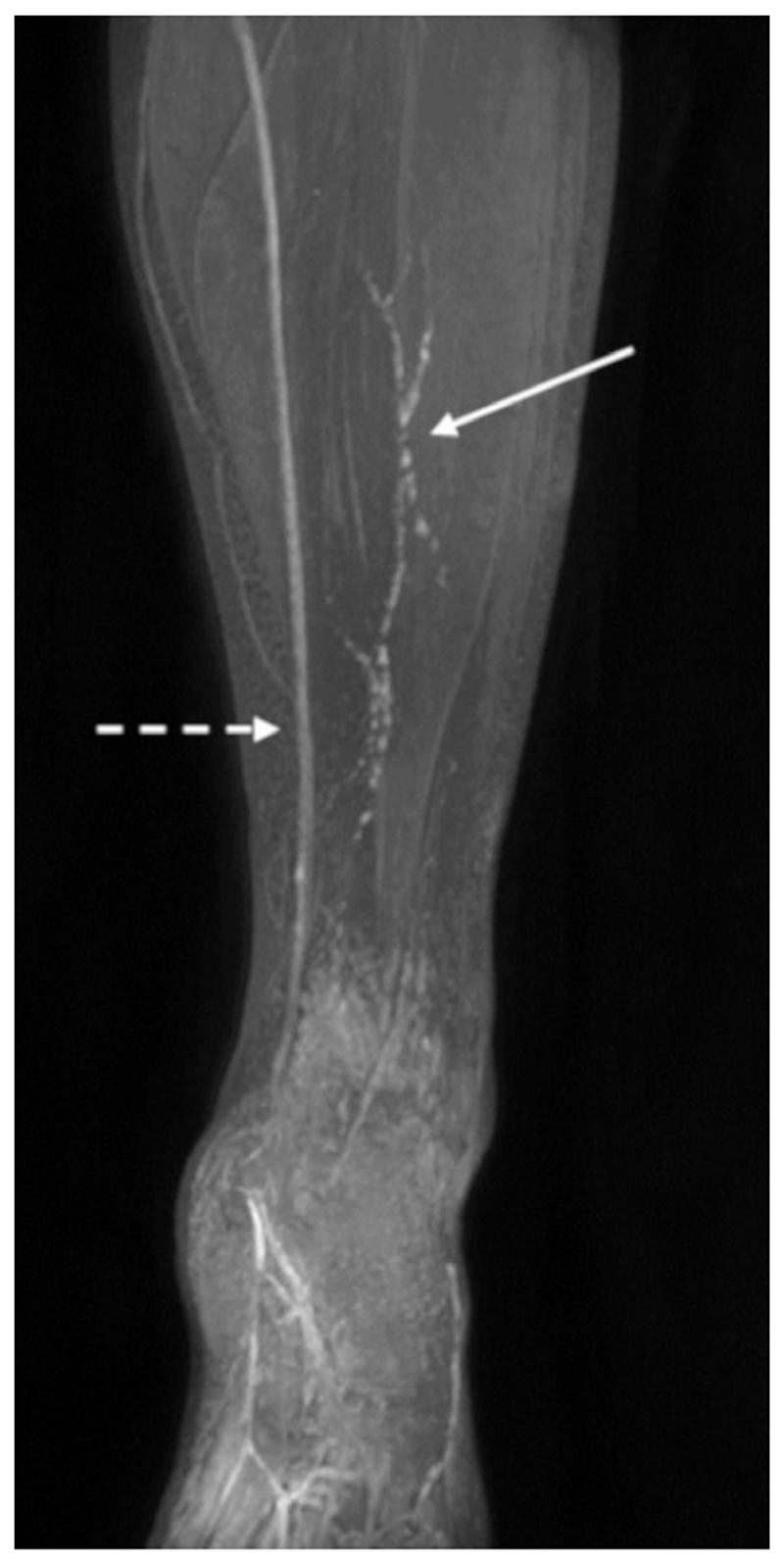
Lower limb MRL of a healthy participant imaged with a fat suppressed contrast-enhanced T_1_ weighted SPGR by St George’s Lymphovascular Research Group. TR/TE = 3.6 / 1.6 msec, flip angle = 12°, reconstructed voxel size = 0.75 × 0.75 × 0.75 mm. This MIP demonstrates thin, discontinuous appearing, lymphatic vessels (solid arrow), as well as larger venous structures (dashed arrow). Image “Lower limb MRL in healthy participant” shared by St George’s Lymphovascular Research Group under the CC BY-SA-4.0 International license (https://creativecommons.org/licenses/by-sa/4.0/). https://commons.wikimedia.org/wiki/File:Lower_Limb_MRL_in_healthy_participant.tif.

MRL has been successfully performed in the arms and legs of participants diagnosed with lymphedema (Figs. 1, 4, 6–7) and healthy participants (Fig. 8).

CE-pMRL is susceptible to the contaminant enhancement of venous structures alongside the lymphatics, however. Despite some authors reporting no difficulty distinguishing enhancing veins from lymphatics based on their appearance, others indicate that venous signal complicates anatomical labeling of enhancing structures.^27,62,63^ Consequently, multiple attempts have been made to proactively reduce the influence of venous enhancement, including: waiting for the venous enhancement to subside^35,53^; collecting a venogram to identify veins^62,64^; injection of ultrasmall superparamagnetic iron oxide (USPIO) for venous suppression^40,57^; or reducing the injected GBCA concentration^8^ (additionally reducing T_2_-related signal loss at the injection site, observed as early as 2006^50^).

Given the short T_1_ time of fat, the majority of T_1_-weighted CE-pMRL studies are performed fat suppressed (Table 2).

### MRL of the Trunk

**Figure 9:**
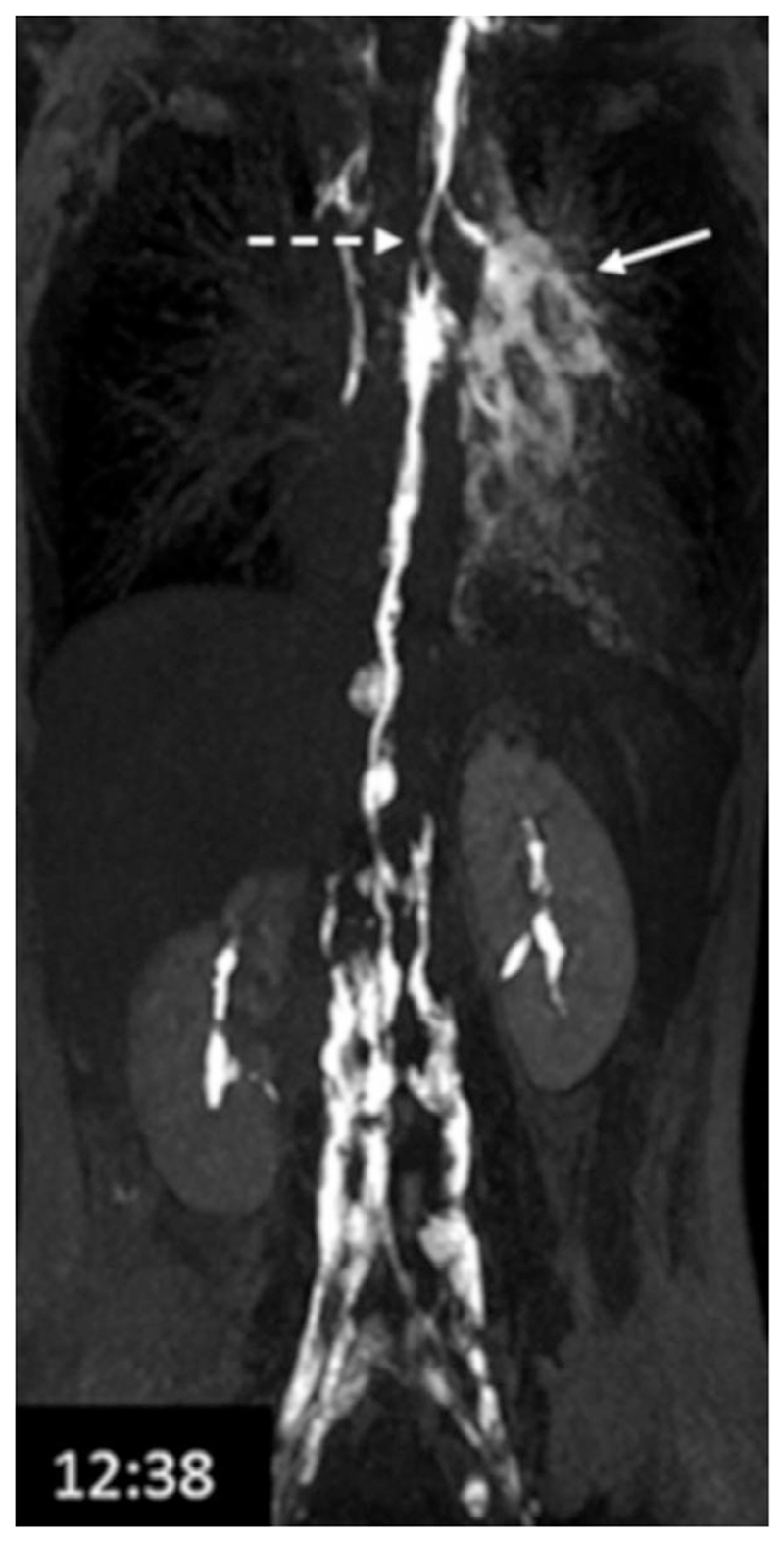
Lymphatic leakage (solid arrow) and thoracic duct narrowing (dashed arrow) identified 12 minutes into imaging of a patient with recurrent chylothorax. Acquired with a fat suppressed SPGR, TR/TE = 4.0/1.9 msec, flip angle = 10°, voxel size = 1.0 × 1.4 × 1.2 mm. Reproduced from Krishnamurthy *et al*.,^47^ with permission.

MRL imaging of peripheral lymphatics has been an active area of research at least since the early 1990s (see, eg, Case *et al.,^65^);* however, imaging the lymphatics of the trunk appears not to have been explored until toward the end of that decade.^66^ Much of the research has focused on imaging the pathway from the two lumbar lymphatic trunks through to the termination of the TD. Figure 3 shows an example of normal appearing TD anatomy, while Fig. 9 demonstrated a narrowed TD and leakage in a patient diagnosed with chylothorax.

The effect of cardiac and respiratory motion is addressed by many studies imaging the LVs in the trunk. Reducing respiratory motion by acquiring data while participants hold their breath was performed in several T_1_-weighted sequences; however, for lengthy T_2_-weighted sequences respiratory gated and cardiac triggered sequences are often preferred (Tables 1–2).

CE studies in the trunk are often acquired after contrast injection into the inguinal LNs, with needle positioning requiring ultrasound or X-ray guidance.^22,47^ Fat suppression techniques were applied in two of four CE studies of the trunk, but only one T_2_-weighted.

### MRL of the Head

**Figure 10:**
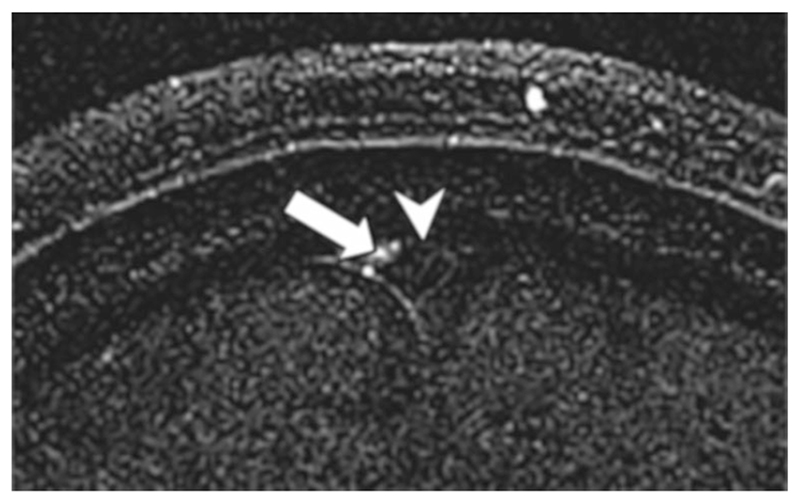
Time of flight (TOF) image in the head of a healthy volunteer showing signal in the meningeal lymphatics (arrow) and low signal in the superior sagittal sinus, SSS (arrow head). Image produced with TR/TE = 30/4.49 msec, flip angle = 10°, voxel size = 0.31 × 0.31 × 1.5 mm, and subtracting images acquired with saturation bands anterior and posterior to the SSS from those acquired with a saturation band only anterior to the SSS. Reproduced from Kuo *et al*.,^19^ with permission.

Only three studies imaging the head were included in this review; however, they demonstrate the ability to detect lymphatic structures in the face, neck, and cranial meninges.^19,44,45^ Two studies perform CE-T_1_ imaging while non-contrast TOF imaging was performed by Kuo et al.^19^ Figure 10 shows an example TOF image in the head. Similar to pMRL, Loo et *al*. reported both enhancement of venous structures, and signal loss at the injection site where GBCA concentration is largest.^44^

MRI studies have begun to investigate the existence and function of a recently hypothesized fluid system in the brain: the glymphatic system.^67,68^ As the name suggests, the glymphatic system (derived from the terms glial and lymphatic) is considered to clear waste from within the brain, as the lymphatic system does throughout the rest of the body, via the cerebrospinal fluid (CSF). Within the glymphatic model, CSF flow is not within an independent vascular system but instead occurs in the perivascular space (unique to neural vasculature) surrounding neural vessels, and is driven by pressure induced from arterial pulsation.^67,68^ The CSF then passes through the brain parenchyma, picking up proteins during this passage, before reaching the perivascular space around the veins and so clearing waste products from the brain. Dysfunction of this drainage pathway has been hypothesized to be linked to neurodegenerative diseases such as Alzheimer’s and Parkinson’s.^69^ The discovery that drainage of waste from the brain occurs not only via perivascular space surrounding veins, but also through a meningeal lymphatic system to the cervical LNs, demonstrates the connection between these systems.^70^ Given the connection to the glymphatic system and hence potential involvement in neurodegenerative disease processes, and the demonstration of MRI to investigate this system in humans, the number of studies reporting meningeal lymphatic MRI is only likely to increase.^19,71^

### Discussion

This review provides evidence that MRI of LVs is viable across the entire body and is capable of demonstrating not only morphological changes with disease, but also altered flow dynamics. There remain no standardized protocols for MRL; however, T_1_-weighted SPGR post intradermal/subcutaneous injection of standard GBCA, and noncontrast T_2_-weighted sequences may be considered standard approaches.

In an attempt to assist readers considering MRL, the remainder of this section is dedicated to the discussion of key technical considerations of MRL protocols and potential avenues of research.

### Spatial and Temporal Resolution

The small size of LVs demands high spatial resolutions for visualization, which limits temporal resolution without advancements in MR hardware (field strength, coil sensitivity, etc.) and k-space sampling techniques. Clinicians and researchers should therefore consider which of these parameters is most important when planning MRL studies.

### Spatial Resolution and LV Visualization

Lymph vessels are typically sub-millimeter in diameter, with only the larger trunks and ducts reaching the millimeter scale.^72^ Compared to 2D, 3D MR acquisitions facilitate thinner slices with less severe partial volume artifacts (also improving SNR for the same effective slice thickness), but increase acquisition times. Gibbs ringing artifacts, series of lines in the image at abrupt signal boundaries such as bright CE LVs and low signal background tissue, may be seen propagating in the slice encoded direction in 3D acquisitions. This is often not observed unless multi-planer reformatting is performed, however.^73^ Regardless of these ringing artifacts, 3D acquisitions are preferable in studies aiming to visualize individual LVs, especially in healthy volunteers, or when estimating LV size.

T_2_-weighted images with relatively low resolutions (~2-3 mm isotropic) appear adequate to identify lymphedematous regions, so improvements of spatial resolution may not be necessary for these already lengthy sequences. If higher resolutions are desired, performing FSE/TSE sequences including a driven equilibrium (DE) pulse may be preferred. In DE sequences, a 90° radiofrequency (RF) pulse at the end of the sequence returns transverse magnetization to the longitudinal plane, recovering transverse magnetization faster than normal T1 relaxation alone. This may therefore accelerate imaging when coupled with reductions in TR, and so be used to offset the increased acquisition time required when increasing image resolution.^74^ Arrivé et al.^28^ and Jeon et al.^56^ employed DE when imaging the limbs of participants diagnosed with lymphedema.

### Lymphatic Contractions and Lymph Transport

Lymphatic contractile frequencies have been estimated at 1.39-6.78 contractions/minute in the TD and ~5 contractions/minute at rest in superficial leg lymphatic collector vessels.^75,76^ These pulsation frequencies are beyond even the most rapid imaging uncovered in the review. Whether it is possible using MRL to measure transient signal changes related to lymph transport, a proxy for lymph pulsation frequency, is yet to be explored, but would require high spatial and temporal resolutions.

The SNR required would also likely need to be improved, especially when imaging at higher resolution (which lowers SNR), in order to detect the signal changes associated with lymphatic propulsion. Imaging at field strengths >3.0 T, and the application of advanced acquisition techniques such as compressed sensing,^77^ would prove beneficial to enable the required spatial resolution, SNR, and accelerated data acquisition.

Physiologically relevant flow measurements have been acquired from MRI datasets, however. Measurements of bulk bolus speed (see Table 4), estimated in three studies using either CE or ASL techniques,^8,9,35^ demonstrate the potential of MRI to monitor lymph flow and may prove beneficial for characterizing lymphatic physiology and diagnosing lymphatic disorders.

### Motion Artifacts

Heavily T_2_-weighted TSE/FSE images remain susceptible to motion artifacts given the long TE and TR required. Imaging lymphatics within the torso has focused on the TD, an area susceptible to the effects of both cardiac and respiratory motion. Proactive steps can be taken to mitigate this issue, including breath-held acquisitions, however Krishnamurthy *et al*. found it necessary to intubate and sedate their participants as age or existing morbidities prevented adequate breath-holds.^47^ Respiratory gating and cardiac triggering have been successfully employed in some of the studies reviewed here, but can elevate total imaging time.^20–23,26,46,47^ Accelerating imaging, via k-space reduction techniques or use of DE for example, may also reduce the likelihood and magnitude of bulk motion artifacts; however, signal loss due to spin dephasing across the lengthy echo train of TSE/FSE will persist.

### Lymph Signal and Background Signal Suppression

MRL image contrast and signal must be sufficient to both identify LVs and distinguish them from other body tissues. Although the SNR in T_2_-weighted images appears much lower compared to CE-T_1_ studies, lymph vessels have been visualized in both.

Image optimization is a nontrivial process and in general MR sequence timing parameters vary as a function of field strength (B_0_): both T_1_ and T_2_ values of tissues are B_0_ dependant, typically increasing and decreasing, respectively, with increases in B0. It is interesting to note the similarity in sequence parameters for both CE-T_1_ and noncontrast T_2_ studies regardless of field strength. This may have arisen as a result of empirically determined optimal sequence parameters; however, this is not commented on within the literature. There is markedly little discussion of optimization of TR/TE within the articles included in this study: adequate image quality with the same protocol despite changes in field strength, and a lack of reported lymph vessel T_1_ and T_2_ times required for robust prospective protocol optimization, may explain the lack of studies documenting image optimization.^61,78^

Flip angle optimization for dynamic CE studies is also nontrivial and requires clear goals; flip angle choice may be different if image contrast or dynamic range are to be optimized for example. Higher flip angles maximize T_1_ weighting, but with an increased potential for generating higher residual fat signals.^79^ Flip angles in the range 10–30° have been reported in CE studies, with none detailing indepth flip angle optimization.

When imaging in fatty regions, fat suppression techniques can improve contrast-to-noise ratio and lymph conspicuity, and techniques insensitive to inhomogeneities in the RF field (also referred to as B_1_ field inhomogeneities) such as Dixon or spectral attenuated inversion recovery are often employed. Dixon methods resilient to B_0_ inhomogeneities have been developed and so may be considered preferable for fat suppression. Acquiring the multiple images required for Dixon studies can increase scan times substantially; however, multi-echo Dixon acquisitions reduce this time penalty.^73,80^ Pieper and Schild performed 3D multi-echo Dixon imaging in participants at 1.5 T. With a resolution of 1.0 × 1.2 × 2.5 mm, they imaged the entire torso with three image stacks requiring 10 seconds each.^48^ Further studies investigating the use of Dixon-based methods across the entire anatomy are required; however, when robust fat suppression is needed Dixon imaging should be considered.

Although no 7.0 T studies of the LVs were uncovered in this review, the feasibility of in vivo human LN imaging at 7.0 T has been demonstrated.^81–83^ Freitag *et al*., performing T_2_-weighted TSE at 7.0 T, highlighted the presence of lymph vessels connected to LN in their high resolution (0.2 × 0.2 × 2 mm) images, emphasizing the utility of ultra-high field strength imaging to generate high-resolution images with sufficient signal to depict both lymphatic nodes and vessels.^83^ Imaging at 7.0 T may also enhance visualization of LVs in healthy limbs which remains difficult at 3.0 T.^41,84^

#### Differentiating Lymphatic and Venous Structures

Differentiating venous and lymphatic structures appears a systemic issue among CE studies. Using vessel morphology or signal enhancement as potential discriminators between LV and veins is commonly reported; however, many authors raise concerns that this approach is insufficient and may decrease the specificity of MRL.^27,62,63^

Acquiring separate venographic images, with or without contrast, may improve visual conspicuity of veins or be used as subtraction masks for MRL data. Image registration may be necessary to reduce potentially confounding subtraction artifacts, however.^8,64^ Noncontrast venograms were produced using balanced steady-state free precession (bSSFP) by Mazzei *et al*.^27^ The large T_2_/T_1_ ratio of lymph raises the possibility of the presence of LVs in these venograms, however, as bSSFP image contrast is T_2_/T_1_ weighted.^85^

The administration of separate USPIO agents in the bloodstream can suppress venous signal by drastically reducing T_2_ times, allowing a selective lymphographic image to be generated.^40,57^ At the time of writing, the agent used in these studies is not licensed for use as an MR contrast agent by the U.S. Food and Drug Administration or the European Medicines Agency. It should be noted that administration of GBCA via skin injection is also considered “off-label”; however, the safety of GBCA delivered by intravenous injection is well established. The risks of GBCA administration (allergy, Gadolinium retention in body tissues, and development of a rare but serious condition in those with renal function: nephrogenic systemic fibrosis) should always be carefully considered prior to injection regardless of route of administration (intravenous or intradermal). Macrocyclic agents such as gadobutrol, gadoteridol, and gadoterate meglumine, should be preferred given their superior safety profiles.^86^

Protocols employing contrast agents to act specifically on venous blood introduce additional safety concerns associated with multiple contrast injections. Large reductions in contrast agent dose, as employed by Borri et *al.,* have the effect of both reducing the potential hazards associated with GBCA delivery and the intensity of venous signal. This, however, has only been demonstrated in a small pilot cohort of subjects.^8^ Alternatively, waiting until the venous signal has decreased, but lymphatic enhancement remains, has been suggested to be a simple and effective solution.^35^ Observation of temporal behavior of lymphatic transport within this wait period may be lost; however, estimations of bulk bolus speed should still be possible.

#### Contrast Agent Delivery

Six GBCA agents, half of which (gadopentetate dimeglumine, gadodiamide, and gadobenate dimeglumine) have had their use restricted within the European Union,^86^ were used within the CE studies. Only one publication investigated the use of different GBCAs (gadoteridol and gadopentetate dimeglumine), concluding that enhancement was equivalent.^44^ This study was conducted in the head and so caution is advised when drawing on these finding when imaging the limbs and trunk. Other articles comment on parameters of GBCAs which may make them optimal for LV studies, such as higher molecular concentration or stronger protein binding.^10,62,87^ A large body of research exists regarding contrast agent use in LN imaging (see, eg, “MR contrast agents in lymph node imaging”^87^), much of which will be relevant to LV imaging; however, specific studies investigating the use of different contrast agents for LV imaging are still required.

#### Injected Solution and Contrast Mobilization

GBCA is most commonly administered undiluted in CE studies; however, as described previously, Borri et al. propose injections heavily diluted with saline such that each milliliter of injected solution contained 0.02 mL of contract agent, 0.1 mL of anesthetic and 0.88 mL saline.^8^ Krishnamurthy et al. also diluted their GBCA with saline when performing intra-nodal injections, using a 1:1 dilution in older patients and a 1:2 GBCA to saline dilution in younger patients. This was performed in order to reduce T2 dephasing effects of the GBCA.^47^ Loo et al. investigated the effect of delivering contrast undiluted vs. diluted and different injection volumes, finding that dilution of GBCA with an equal volume of sterile water, and smaller injections of 0.3–0.5 mL per injection, provided optimal lymphatic enhancement in the head.^44^

Massage proximal to the contrast injection site is common after intradermal/subcutaneous contrast injection, but there is no clear consensus as to how, or if, to add an intervention to improve contrast mobilization into the lymphatics. Loo et al. demonstrated that repeated massage extended the time over which LV enhancement was sustained and produced additional signal peaks,^44^ perhaps due to increased interstitial pressure from the massage driving contrast into the lymphatics.^88,89^ Pieper and Schild requested that participants move their limbs after contrast injection, presumably in an attempt to increase contrast uptake, a method employed regularly for ICG and LS.^48^ The extent to which this changed contrast uptake was not explored, however.

While clear that standard GBCAs can be used for LV imaging, variable number of injection sites, injected volume, and GBCA formulation have been employed, and more research is required before an optimal injection protocol can be recommended. A systematic exploration of the effect of different injection and intervention (eg, massage) protocols on contrast uptake, study repeatability, and to what extent subtle lymphatic insufficiencies could be masked, is needed.^90–92^

#### Quantitative Analysis

MRL has been shown to visualize structural abnormalities of the lymphatic system, with additional quantitative analyses differentiating healthy and abnormal groups. Common measurements include counting visible LVs, estimating vessel diameter, and recording signal enhancement characteristics.

Many studies use the contralateral limb as an internal control in both qualitative and quantitative studies. The results of such comparisons should be approached with caution as abnormal imaging signs within the contralateral limb have been observed.^26,93^ Enrollment of a healthy matched control cohort would reduce the risk of such confounders.

#### Vessel Size

The thickness of the TD has been estimated by multiple authors, often enrolling participants with nonlymphatic specific abnormalities such as liver malignancy and a Fontan circulation,^20,26^ with diameters in the region of 1–7 mm observed. Larger peripheral LVs in individuals with lymphatic disease have also been commonly observed compared to healthy controls.

Regardless of anatomy, absolute measurements of LV diameter will be prone to error when voxel sizes are similar to, or greater than, the vessel size. Acquiring higher spatial resolution images will improve the accuracy with which LV size can be estimated. For large field of view studies, ~ 1 mm^3^ voxels may be approaching the maximum feasible resolution for current clinical MR systems. Imaging at higher field strengths and employing acceleration techniques such as compressed sensing and multiband RF imaging to improve image resolution should be considered if more representative estimates of vessel size are required.

#### Lymph Flow and Contrast Distribution

Time to peak lymphatic enhancement has been estimated in multiple CE studies. These values will likely depend on measurement location and injection protocol (eg, contrast agent, dose, massage, etc.), and are hence difficult to compare directly.

LS has long been used to estimate lymph drainage by determining tracer uptake in the LNs.^94^ Although not explored in any of the articles reviewed here, similar measurements may be possible via MRL with T_1_ measurements in the LVs or LNs yielding estimates of local GBCA concentration. This requires that sufficiently low injected GBCA concentrations and high flip angles are employed to ensure a linear relationship between image signal and 1/T_1_ is maintained. Estimates of T_1_ will be affected by factors such as fluid flow and diffusion, partial volume, changes in local proton-density, and field inhomogeneities, and so will require good experimental design.^95,96^

Lymph speed has been estimated in the limbs by three studies employing different MRL methods and analysis models. With an ASL based approach, measuring signal as a function of post-labeling delay time, lymph speed in the arm was estimated from signal in a downstream LN in the arms of BCRL patients.^9^ Imaging the leg, Liu *et al*. recorded lymph speeds consistent with those achieved with ASL by measuring LV length on CE images and calculating speed as enhanced vessel length divided by the acquisition time.^35^ Borri *et al*. recorded slightly higher speeds in their single participant with BCRL.^8^ A five-parameter modified logistic model was employed to fit signal enhancement, with one parameter representing the GBCA arrival time. It is interesting to note that despite the methodological differences, reported speeds are similar (~0.5–2 cm/minute) for affected limbs across these studies.

#### Is MRL Superior to ICG and LS?

LS is currently considered the clinical gold standard for diagnostic lymphatic imaging. Given the sparsity of studies comparing techniques directly, or high-level evidence such as meta-analyses, it is difficult to conclude which technique is superior. However, it is interesting to note that all studies within this review comparing MRL and LS report improved LV visualization in the limbs with MRL.^5,6,34,43^ The superiority of MRL may also become more evident with further optimization.

It is perhaps more pertinent to comment on the complementary nature between MRL, LS and ICG lymphography, and a combination of MRL with either may deliver a more complete understanding of lymphatic anatomy and physiology than MRL alone. While ICG yields high spatial and temporal imaging of superficial lymph vessels, which may lead to estimations of vessel contraction frequency, MRL facilitates evaluation of the lymphatic system over large anatomical regions and can image both superficial and deep lymphatic structures such as the TD.^26,52^ LS, while lacking spatial and temporal resolution, is readily quantifiable to estimate tracer clearance and hence lymphatic transport. Studies of lymphatic transport by MRI are being performed, both noncontrast (using an ASL approach) and contrast-enhanced, but further research is required before MRL studies of lymphatic transport can be interpreted with a high degree of confidence, and routinely implemented.

### Conclusion

In conjunction with basic biological research and imaging techniques such as ICG lymphography, LS, and histology, MRL can become a powerful tool in gaining a more detailed understanding of the complexities of the lymphatic system. The potential for MRL research to directly influence clinical practice in diseases of the lymphatic system was recently demonstrated in an article reporting 92% sensitivity in identifying lymphedema with MRL alone.^93^ Studies investigating factors such as: the influence of administered contrast agent formulation and massage on contrast uptake characteristics; optimal imaging parameters for T_2_-weighted depiction of LVs; and relevance of quantitative image markers such as estimates of lymph speed and vessel size to lymphatic function, are still required to truly unlock MRLs diagnostic and prognostic potential.

## Supplementary Material

Supplementary file

## Figures and Tables

**Table 1:** Noncontrast Lymphatic Sequences, Excluding Those Acquired for Node Visualization

Anatomical Region	Field Strength (T)	Sequence Variant	TR/TE (msec)	Flip Angle (°)	Acquisition Time (minutes: seconds)	Resolution		Additional Parameters			Source
						In-Plane Matrix	Reconstructed Voxel (mm)	Fat Suppressed?	Motion Reduction	Other	
Head and Neck	3.0	Flow weighted TOF	30/4.49	10	—	160 × 160	0.31 × 0.31 × 1.5			Anterior and posterior saturation bands, NSA = 10	^19^
Torso	1.5	3D FSE	3000–6000/500	—	—	320 × 320	1.1 × 1.1 × 2.0		Respiratory gated	Partial Fourier	^20^
Torso	1.5	3D TSE	2500/650	140	2–5	256 × 256	1.1 × 1.1 × 1.1		Respiratory navigated, cardiac gated		^21^
Torso	1.5	3D TSE	2500/650	140	2–5	256 × 256	1.2 × 1.2 × 1.2		Respiratory navigated, cardiac gated		^22, 23^
Torso	3.0	—	2000–4500/550–750	—	5–10	320 × 256	1.2-1.4 × 1.5 –1.8 × 1.0–2.0				^24^
Torso	3.0	3D TSE	3000/600	110	10:51	—	1.39 × 1.39 X3.0	Yes		Anterior and posterior saturation bands, NSA = 2	^25^
Torso	3.0	3D FSE	2830/649	125	—	448 × 448	0.9 × 0.9 × 0.8		Respiratory gated		^26^
Peripheral	1.5	T_2_/T_1_ weighted 3D bSSFP	4.0/1.9	—	—	224 × 192	1.8 × 2.1 × 2.0	Yes	ECG triggered	Partial Fourier	^27^
Peripheral	1.5	3D FSE	4000/884	90	3-5	512 × 288	0.8 × 1.4 × 0.8-1.4			Driven equilibrium	^28^
		3D Dixon	4233/76	—	3:30	320 × 192	1.2 × 2.0 × 6.0			—	
Peripheral	1.5	3D FSE	4000/884	90	—	—	—				^29^
		3D Dixon	4233/76	—	—	—	—				
Peripheral	1.5	3D TSE	2000/694	180	4:04	256 × 256	2.0 × 1.9 × 1.7				^30–33^
Peripheral	1.5	3D TSE (SPACE)	4000/221	120	—	—	1.0 × 1.4 × 1.5	Yes		Parallel imaging acceleration factor = 3	^34^
Peripheral	3.0	3D TSE	2820/740	—	—	240 × 190	1.5 × 1.5 × 2.0			Partial Fourier	^35–38^
Peripheral	3.0	3D TSE	3600/80	90	—	320 × 304	1.15 × 1.05 × 5	Yes			^39^
Peripheral	3.0	3D TSE	3000/600	110	10:51		1.39 × 1.39 × 3.0	Yes		Anterior and posterior saturation bands, NSA = 2	^25^
Peripheral	3.0	3D TSE	2500/650	—	—	—	1.6 × 1.9 × 2.8	Yes		Partial Fourier, parallel imaging acceleration factor =1.9	^40^
Peripheral	3.0	TSE	3690 / 80	—	—	352 × 256	0.9 × 1.2 × 6.0				^41^
Peripheral	3.0	3D FSE	2830 / 649	125	—	448 × 448	0.9 × 0.9 × 0.8				^26^
Peripheral	3.0	STIR	5940/90	120	—	—	1.8 × 1.3 × 3			Parallel imaging acceleration factor = 2, TI = 180 msec	^42^
Peripheral	3.0	3D RARE	5940/90	120	—	—	1.8 × 1.3 × 3			Parallel imaging acceleration factor = 2, TI = 180 msec	^43^
Peripheral	3.0	RARE	—	—	—	—	—				^5^
Peripheral	3.0	Flow weighted ASL	–/4	—	40	—	3×3×5	Yes		TI = 3500-10000 msec, NSA = 8^a^, Partial Fourier, parallel imaging acceleration factor = 2	^9^

All studies are T_2_-weighted unless otherwise stated. Where field of view and matrix are present, but voxel sizes not stated, calculated voxel sizes are displayed. The Fat Suppressed column indicates if a fat suppression pre-pulse was used; Dixon imaging or water frequency selective excitations are not considered as such.

a Values quoted from imaging affected participants. Variations in protocol between affected and unaffected participants can be seen in the original article.

TOF = time of flight; RARE = Rapid acquisition with relaxation enhancement; STIR = short tau inversion recovery; TSE/FSE = turbo/fast spin echo; bSSFP = balanced steady-state free precession; ASL = arterial spin labeling; NSA = number of signal averages.

**Table 2:** Contrast-Enhanced Studies

Anatomical Region	Field Strength (T)	Sequence Variant	TR/TE (msec)	Flip Angle (°)	Acquisition Time (sec)	Resolution		Additional Parameters			Source
						In-Plane Matrix	Reconstructed Voxel (mm)	Fat Suppressed?	Motion Reduction	Other	
Head and Neck	1.5	3D SPGR (SMMT)	27.5/8.5	50	—	512 × 192	—				^44^
Head and Neck	1.5	—	5.01/1.03	30	—	195 × 256	1.5 × 0.9 × 1.3				^45^
Torso	1.5	3D SPGR	4.6/1.2	15	32-42	256 × 128	1.7 × 3.0-3.4 × 2.0	Yes	Breath-hold		^46^
Torso	1.5	3D SPGR (THRIVE)	4/1.9	10	20-30	—	0.65-1 × 0.65-1, 1-1.3	Yes	Breath-hold	Partial Fourier, parallel imaging acceleration factor = 2–4	^47^
Torso	1.5	MRA (TWIST)	3/1	25	900	320 × 240	1.2 × 1.2 × 1.2		Navigator gated		^22, 23^
		3D IR-FLASH	300/1.5	20	—	320 × 240	1.2 × 1.2 × 1.2				
Torso	1.5	3D Dixon	“shortest”/1.8, 4.0	15	10 per stack (3 stacks required to image entire torso)	—	1×1×1		Breath-hold	Parallel imaging acceleration factor = 1.65	^48^
Peripheral^a^	1.5	3D GRE	5.1/1.4	30	-180^b^	256 × 192	-0.7 × 0.6 × 1.4^b^			NSA= 2	^10^
Peripheral	1.5	3D GRE	4.8/1.4	30	—	—	—				^49^
Peripheral	1.5	3D SPGR (VIBE)	3.4/1.47	25	44	448 × 448	2.2 × 1.1 × 1.5				^30, 33^
Peripheral	1.5	3D SPGR (VIBE)	3.58/1.47	35	100	448 × 448	1.2 × 1.1 × 1.2				^31, 32^
Peripheral	1.5	3D SPGR (FLASH)	5.1/1.23	25	31	448 × 448	2.0 × 1.0 × 1.0				^50^
Peripheral	1.5	3D SPGR	6.14/2.77	12	78	—	1×1×1	Yes			^8^
Peripheral	1.5	3D SPGR	5.0/2.1	25	250	448 × 320	1.0 × 1.4 × 2.8	Yes			^27^
Peripheral	1.5 and 3.0		5.60/1.86	—	—	—	– × – × 0.5				^51^
Peripheral	3.0	3D GRE	5.7/2.5	70	120	380 × 70	1.1 × 5.7	Yes			^52^
Peripheral	3.0	3D SPGR (FLASH)	4.13/1.47	25	149	448 × 448	0.8 × 0.8 × 0.8	Yes		Parallel imaging acceleration factor = 3	^42^
Peripheral	3.0	3D SPGR (THRIVE)	3.5/1.7	25	40	300 × 256	1.5 × 1.2 × 1.2	Yes		NSA = 2	^35–38^
Peripheral	3.0	3D SPGR (THRIVE)	3.5/1.7	25	180	300 × 256	1.4 × 0.5 × 0.5	Yes			^53^
Peripheral	3.0	3D SPGR (THRIVE)	23/2.1	15	180	760 × 720	0.5 × 0.5 × 1.3			Parallel imaging acceleration factor = 2, NSA = 2	^39^
Peripheral	3.0	3D SPGR (THRIVE)	6.4/1.7	100	130	300 × 256	1.2 × 1.2	Yes			^41^
Peripheral	3.0	3D SPGR (THRIVE)^d^	2820/740	25	60	240 × 190	1.5 × 1.0 × 1.0	Yes		NSA = 2	^54^
Peripheral	3.0	3D SPGR	3.5/1.7	25	180	750 × 640	1.2 × 0.5	Yes			^55^
Peripheral	3.0	3D SPGR (FLASH)	3.5/1.3	14.9	70	228 × 202	1.0 × 1.4 × 1.2	Yes		Parallel imaging acceleration factor = 2	^34^
Peripheral	3.0	3D SPGR (FLASH)	4.13/1.47	25	149	448 × 448	0.8 × 0.8 × 0.8	Yes		Parallel imaging acceleration factor = 3	^43^
Peripheral	3.0	3D SPGR	4.13/1.47	25	149	448 × 448	0.8 × 0.8 × 0.8	Yes		NSA = 3	^5^
Peripheral	3.0	3D FSE (VISTA)	350/17	—	227	—	1×1×1	Yes		Parallel imaging acceleration factor = 2–2.5	^56^
		3D proton density weighted FSE (VISTA)	1400/40	—	284		1×1×1	Yes		Parallel imaging acceleration factor = 2–2.5; driven equilibrium	
Peripheral	3.0	3D Dixon	–/optimized, optimized^c^	20	60-90	-220 × 220^b^	-1.4 × 1.4 × 1.8^b^			Venous suppression with USPIO	^57^
Peripheral	3.0	3D Dixon	“shortest”/optimized, optimized^c^	20	—	—	—			Venous suppression with USPIO	^40^

All studies are T_1_-weigh red unless otherwise stated. Where field of view and matrix are present, but voxel sizes not stated, calculated voxel sizes are displayed. The Fat Suppressed column indicates if a fat suppression pre-pulse was used; Dixon imaging or water frequency selective excitations are not considered as such.

SPGR = spoiled gradient echo; GRE = gradient echo; TSE/FSE = turbo/fast spin echo; NSA = number of signal averages; MRA = magnetic resonance angiography; USPIO = ultra-small super-paramagnetic iron oxide; SMMT = spectral-spatial excitation magnetization transfer.

a Study included a single participant imaged in the torso which is not detailed here.

b Varies with anatomy; representative value given.

c Echo time optimized per participant. Note also that for MRL without the addition of an USPIO TR/TE1/TE2 = 4.4-4.5/1.2-1.5/2.4-2.7 msec.

d Described within the source as T_1_ weighted despite the sequence parameters.

**Table 3:** Contrast Injection and Massage Protocols in Contrast-Enhanced Studies

Anatomical Region	Field Strength (T)	Contrast Agent(s)	#of Injections	Location of Injection	Injection Solution		Injection Volume (Per Site)	Massage of Injection Site	Source
					GBCA Vol.	Other Added			
Head and Neck	1.5	Gadoteridol	Variable	Variable	Variable	Variable	≤1 mL	Variable	^44^
		Gadopentetate dimeglumine							
Head and Neck	1.5	Gadopentetate dimeglumine	5	Bilateral submucosa of the pharyngeal recess	4.5 mL	0.5 mL LH (2%)	1 mL	1 minute	^45^
Torso	1.5	Gadopentetate dimeglumine	2	Peri areolar	1.0 mL	0.25 mL LH (1%)	0.5 mL	—	^46^
Torso	1.5	Gadopentetate dimeglumine	2	Inguinal LNs	0.1 mmol/kg	Equal volume of saline^b^	—	—	^47^
Torso	1.5	Gadopentetate dimeglumine	2	Inguinal LNs	Variable	—	2-8 mL	—	^22, 23^
Torso	1.5	Gadobutrol	4	Digital webspaces	6.0 mL	2 mL saline (post 0.2 mL 1% MH)	1 mL	—	^48^
Peripheral	1.5	Gadoterate meglumine	5	Webspaces + medial to 1st distal metatarsal	4.5 mL	0.5 mL LH (2%)	1 mL	2 minutes	^10^
Peripheral	1.5	Gadodiamide	5	Digital webspaces + medial to 1st proximal phalanx	4.5 mL	0.5 mL MH (1%)	1 mL	1 minute^a^	^50^
Peripheral	1.5	Gadodiamide	5	Digital webspaces + medial to 1st proximal phalanx	18 mL	2 mLMH (1%)	2 mL	1 minute^a^	^33^
Peripheral	1.5	Gadodiamide	5	Digital webspaces + medial to 1st proximal phalanx	0.1 mmol/kg	2 mLMH (1%)	≤1.8 mL	1 minute^a^	^30^
Peripheral	1.5	Gadodiamide	5	Digital webspaces + medial to 1st proximal phalanx	9.0 mL	1 mLMH (1%)	2 mL	—	^31^
Peripheral	1.5	Gadobutrol	5	Digital webspaces + dorsal area of foot	4.5 mL	0.5 mL LH (2%)	1 mL	5 minutes	^49^
Peripheral	1.5	Gadoteridol	5	Digital webspaces + medial to 1st proximal phalanx	18 mL	2 mLMH (1%)	2 mL	—	^32^
Peripheral	1.5	Gadoteridol	4	Digital webspaces	0.9 mL^c^	0.1 mLLH (1%)	1 mL	—	^8^
					0.02 mL^c^	0.1 mL LH (1%) + 0.88 mL saline			
Peripheral	1.5	Gadobenate dimeglumine	4	Digital webspaces	0.1 mL/kg	1 mL LH (2%)	≤1 mL	—	^27^
Peripheral	1.5 and 3.0	Gadodiamide	Variable	Variable	12-20 mL	4 mL LH (2%)	Variable	0.5 minute	^51^
Peripheral	3.0	Gadobenate dimeglumine	4	Digital webspaces	15 mL	1.5 mLLH (1%)	0.7-0.8 mL	—	^35^
Peripheral	3.0	Gadobenate dimeglumine	—	—	—	—	0.7-0.8 mL	—	^36^
Peripheral	3.0	Gadobenate dimeglumine	4	Digital webspaces	8.0 mL	1 mLMH (1%)	1.1 mL	—	^38^
Peripheral	3.0	Gadobenate dimeglumine	4	Digital webspaces	—	10:1 ratio GBCALH (1%)	1 mL	0.5 minute	^39^
Peripheral	3.0	Gadobenate dimeglumine	1	Base of scrotum	—	10:1 ratio GBCALH (10%)	0.5 mL	0.5 minute	^55^
Peripheral	3.0	Gadobenate dimeglumine	4	digital webspaces	—	—	0.7-0.8 mL	—	^37^
Peripheral	3.0	Gadobenate dimeglumine	2	2nd and 4th digital webspaces	0.8 mL	0.2 mL scandinibsa^d^	1 mL	—	^52^
Peripheral	3.0	Gadobenate dimeglumine	4	Digital webspaces	5.0 mL	1 mL LH (1%)	1 mL	—	^57^
		Ferumoxytol	—	Intravenous	5 mg/kg	Saline dilution to 60 mL total volume	60 mL		
Peripheral	3.0	Gadobenate dimeglumine	4	Digital webspaces	5.0 mL	1 mL LH (1%)	1 mL	—	^40^
		Ferumoxytol	—	Intravenous	5 mg/kg	Saline dilution to 60 mL total volume	60 mL		
Peripheral	3.0	Gadopentetate dimeglumine	3	Digital webspaces	5.5 mL	0.5 mL MH (1%)	1 mL	0.5 minute	^53^
Peripheral	3.0	Gadopentetate dimeglumine	3	1st three digital webspaces	5.4 mL	0.6 mL MH	1 mL	2 minutes	^42^
Peripheral	3.0	Gadopentetate dimeglumine	3	1st three digital webspaces	5.4 mL	0.6 mL MH	1 mL	2 minutes	^43^
Peripheral	3.0	Gadopentetate dimeglumine	3	1st three digital webspaces	-	MH	1 mL	2 minutes	^5^
Peripheral	3.0	Gadopentetate dimeglumine	4	Digital webspaces	15 mL	1.5 mLLH (1%)	0.7-0.8 mL	2 minutes	^41^
Peripheral	3.0	Gadopentetate dimeglumine	3	Three finger digital webspaces	5.5 mL	0.5 mL MH (1%)	1 mL	0.5 minute	^54^
Peripheral	3.0	Gadobutrol	3	1st three digital webspaces	0.1 mmol/kg	0.5 mLLH (1%)	≤2 mL	1 minute	^56^
Peripheral	3.0	Gadobutrol	4	Digital webspaces	4.5 mL	0.5 mL LH	1 mL	2 minutes	^34^

Standard concentrations of each agent, in mol/L, are: gadopentetate dimeglumine, 0.5; gadoteridol, 0.5; gadobutrol, 1.0; gadoterate meglumine, 0.5; gadodiamide, 0.5; gadobenate dimeglumine, 0.5.

LH = lidocaine hydrochloride; MH = mepivacaine hydrochloride.

a Repeated between or during data acquisition.

b Dilution with saline doubled in “younger” participants.

c Per injection site.

d Active ingredient mepivacaine hydrochloride.

**Table 4:** Summary of Commonly Reported Findings Presented in Each Study

Anatomical Region	Field Strength (T)	Subjects	Qualitative Measurements			Quantitative Measurements			Source
			Dermal Rerouting?	Fluid Accumulation?	Other	Size	Signal	Other	
Head and Neck	1.5	Healthy volunteers			LV and blood signal intensity with protocol variations		LN enhancement ratio vs. time	LN count	^44^
Head and Neck	1.5	Nasophary ngeal carcinoma							^45^
Head and Neck	3.0	Healthy volunteers			Presence of meningeal LVs				^19^
Torso	1.5	Healthy volunteers				LN diameter: mean = 4.1 ± 2.2 mm (sentinel node), = 4.3 ± 0.8 (distal nodes)	Normalized LV and LN signal vs. time	LN count	^46^
Torso	1.5	Liver disease and malignancy				TD diameter: mean = 4.23 ± 1.76 mm in affected participant, 3.74 ±0.81 mm in healthy volunteers			^20^
Torso	1.5	Functional singleventricle palliation surgery		Yes	Collateral LVs	TD diameter: range = 1.3–7.2 and 1.7–2.6 mm in surgical and non-single-ventricle heart disease participants			^21^
Torso	1.5	Central conducting lymphatic anomalies		Yes	Collateral LVs, retrograde flow, LV occlusion and lymph leakage				^47^
Torso	1.5	Congenital heart disease			Retrograde flow				^23^
Torso	1.5	Plastic Bronchitis			LV occlusion and retrograde flow				^22^
Torso	1.5	Chylous effusions		Yes	Retrograde flow and lymph leakage				^48^
Torso	3.0	Healthy volunteers							^24^
Torso	3.0	BCRL		Yes		Max LV area: 17.2 ± 15.6 mm^2^ in the affected side of participants, 8.7 ± 2.1 and 8.7 ± 2.8 mm^2^ in the left and right side of healthy volunteers	Lymphatic SNR		^25^
Torso	3.0	Fontan circulation		Yes	Collateral LVs	TD: diameter: mean = 2.7 ± 1.1 mm in both affected and unaffected participants. TD relative length: mean = 1.12 ± 0.09 mm in affected, 1.05 ± 0.04 mm in unaffected volunteers			^26^
Peripheral	1.5	Lymphoceles		Yes	LV leakage		LV and LN SNR vs. time		^10^
Peripheral	1.5	Lower limb lymphedema	Yes		Collateral LVs	Max LV diameter: 5 mm	Time to maximal LV, LN and venous signal intensity		^30^
Peripheral	1.5	Lower limb lymphedema	Yes			Max LV diameter: 5 mm	Time to maximal LV, LN and venous signal intensity		^50^
Peripheral	1.5	Lymphoceles	Yes	Yes	LV leakage	Max LV diameter: 5 mm	LV and vein signal vs. time		^33^
Peripheral	1.5	Lower limb lymphedema	Yes	Yes	Collateral LVs	LV diameter: range =1–5 mm	LV, LN and vein SNR vs. time		^31^
Peripheral	1.5	Lower limb lymphedema		Yes	Relative LV count				^49^
Peripheral	1.5	Lower limb lymphedema		Yes	LV Occlusion, collateral LVs	LV diameter: range =1–5 mm	LN enhancement vs. time		^32^
Peripheral	1.5	Lower limb lymphedema		Yes	Honeycomb pattern			LN count	^28^
Peripheral	1.5	BCRL			Visibility of injection site and blood signal intensity with protocol variations		LV and vein signal vs. time	LV speed = 9-7, 2.1 cm/ minute in a healthy and affected limb	^8^
Peripheral	1.5	Upper or Lower limb lymphedema and BCRL	Yes	Yes	Honeycomb pattern, collateral LVs	Mean LV diameter: 2.2 ± 0.5 and 1.5 ± 0.2 mm in affected and unaffected limbs, respectively	LV SNR vs. time	LV count	^27^
Peripheral	1.5 and 3.0	Cervical cancer							^51^
Peripheral	3.0	Lymphedema, lymphoceles or LV transplant	Yes	Yes			LV and venous SNR and CNR		^42^
Peripheral	3.0	Lymphedema or LV transplant	Yes						^43^
Peripheral	3.0	Lower limb lymphedema	Yes	Yes		LV diameter: range = 1.2-8 mm	LN SNR vs. time	LV and LN count: LV range 1 – “numerous”, lymph speed: range = 0.3-1.48 cm/minute	^35^
Peripheral	3.0	Lower limb lymphedema	Yes	Yes	Honeycomb pattern, collateral LVs	Max LV diameter: 4.28 ±1.53 and 3.41 ± 1.05 in T_2_-w and CE-T/ images, respectively	LV SNR and CNR	LV count: mean = 6.82 ± 5.10, 4.88 ± 4.18, in T_2_ and CE-Ti weighted images, respectively)	^38^
Peripheral	3.0	Lower limb lymphedema		Yes		LV diameter: range = 0.5–8 mm		LV count: range = 0 to “numerous”	^36^
Peripheral	3.0	Lower limb lymphedema	Yes			LV diameter: median = 3.41 ± 1.4, 2.49 ± 0.79 mm and 2.11 ± 1.25, 1.29 ± 0.35 mm in affected and unaffected calf and thigh, respectively		LV count: median = 7, 10 and 5, 5 in unaffected and affected calf and thigh, respectively	^53^
Peripheral	3.0	Lower limb lymphedema	Yes						^5^
Peripheral	3.0	Lower limb lymphedema	Yes		Honeycomb pattern	LN diameter	LN enhancement ratio vs. time		^41^
Peripheral	3.0	Upper or Lower limb lymphedema	Yes		More LV observed in T_1_ vs. PD weighted images				^56^
Peripheral	3.0	Upper or lower limb lymphedema	Yes		Reduction in venous signal with USPIO injection		LV signal and LV to muscle contrast ratio as a function of TE (signal reduced by 45% and contrast by 21 % in long TE sequence)		^40^
Peripheral	3.0	Upper or lower limb lymphedema	Yes		LV location				^52^
Peripheral	3.0	Upper limb lymphedema	Yes			LV diameter: mean = 3-06 ± 0.78 vs. 1.98 ± 0.30 mm in affected participant vs. healthy controls			^34^
Peripheral	3.0	BCRL						Lymph Speed = 0.48 ± 0.15 and 0.58 ± 0.16 cm/ minute in affected vs. unaffected cases	^9^
Peripheral	3.0	BCRL	Yes	Yes	LV leakage	LV diameter: range = 0.5–5 mm		LV count: median = 4	^37^
Peripheral	3.0	BCRL		Yes					^29^
Peripheral	3.0	BCRL			Collateral LVs	Max LV area: 12.9 ± 6.3 mm^2^ in the affected side of participants, 8.8 ± 4.2 and 8.4 ± 1.6 mm^2^ in the left and right side of healthy volunteers	Lymphatic SNR		^25^
Peripheral	3.0	BCRL	Yes	Yes	Honeycomb pattern, LV leakage	LV diameter: mean = 1.73 ± 0.24, 0.65 ± 0.36 mm in affected participant vs. healthy controls			^54^
Peripheral	3.0	Inguinal lymphatic vessel leakage		Yes	Honeycomb pattern, LV leakage		SNR in LV leakage site and LNs	Leaking LV count: range = 1–5 (median = 2)	^39^
Peripheral	3.0	Genital lymphedema	Yes	Yes			LN signal vs. time	LV count	^55^
Peripheral	3.0	Fontan circulation	Yes						^26^
Peripheral	3.0				LV and blood signal intensity with protocol variations				^57^

The subject column details the affected cohort, except when only healthy volunteers were enrolled. All articles include some reference to the presence or morphology of LVs (eg, shape, dilation, and tortuosity). Note that, despite not being specifically lymphatic, the presence of a honeycomb pattern in the soft tissue is included here given the frequency of reporting.

LV = lymphatic vessel; LN = lymph node; SNR = signal-to-noise ratio; CNR = contrast-to-noise ratio; BCRL = breast cancer related lymphedema; USPIO = ultrasmall superparamagnetic iron oxide.
