# Supplementary material for: Systematic Review of Magnetic Resonance Lymphangiography From a Technical Perspective
Source: J Magn Reson Imaging. Author manuscript; Available in PMC 2021 Sep 9. (PMC7611641; doi:10.1002/jmri.27542)
Supplement: Supplementary file [file EMS133895-supplement-Supplementary_file.docx]

Systematic Review Quality Assessment Protocol

1. Introduction

A protocol has been produced with heavy reliance on the NIH quality assessment tool for case series studies [1] and Downs and Blacks quality checklist for health care intervention studies [2]. It has been supplemented and adapted to be most suited to research / original articles of human participants undergoing MRI to investigate the lymphatics vessels and wider lymphatic system.

2. Method
2.1 Quality Criteria Outline
The assessment criteria consist of 9 questions (Supplementary Table 1) with differing weightings. Each article is to be assessed against all questions where relevant, making the maximum score for any one article 14. When a question, or part of the question, does not apply it is scored with ‘Not Applicable’ (N/A). For example, if a study includes no contrast agent, Q4 and part of Q6 are not applicable and the marks for these questions are to be excluded from the final tally so the maximum mark immediately becomes 12. Additionally, many articles include no numerical results and are purely descriptive. In this case Q7 and Q8 are also considered ‘Not Applicable’. The marks for those questions scored as N/A are excluded from the final tally of maximum available marks for each article. Additionally, when a paper does not provide sufficient detail to answer a question, the score ‘Unable to Comment’ (UTC) is given. No marks are awarded for that question; however, the marks are still considered to have been available and so are included in the final tally of maximum possible marks. Using this scoring system papers can be ranked according to how much or little detail they provide and gives an overview of how useful the various papers can be.

**Supplementary Table 1.** The purpose designed assessment tool consist of nine questions with different weighting. The maximum mark a study can potentially score is 14 if contrast agent is used, or 12 for non-contrast studies.

|  |  | Max Score | |
| --- | --- | --- | --- |
|  |  | CE-MRL | Non-contrast |
| Q1. | Was the patient/volunteer population well described and selection criteria clear? | 2 | 2 |
| Q2. | Were case and control groups enrolled and were they matched? | 2 | 2 |
| Q3. | Was the imaging procedure clearly described and did it remain constant across the sample group? | 2 | 2 |
| Q4. | **CE-MRL only** - was the injection protocol clearly described? | 1 | N/A |
| Q5. | Were the outcome measures clearly defined, implemented and reported consistently across all study participants? | 1 | 1 |
| Q6. | Was participant compliance high and for **CE-MRL only** were side-effects documented? | 2 | 1 |
| Q7. | Where relevant, were statistical/numerical analyses/measurements well described and appropriate? | 1 | 1 |
| Q8. | Where relevant, were numerical results clearly displayed and include estimates of variability? | 1 | 1 |
| Q9. | Were imaging issues/practicalities well documented and were they actively addressed? | 2 | 2 |
|  | *Max Total Marks* | *14* | *12* |

As can be seen from the above, the largest weighting is applied to the clarity of the imaging technique (Q3 and Q9), including injection protocol (Q4) and, and participant selection (Q1 and Q2) and compliance (Q6). This reflects the purpose of the review: to discuss the MRL imaging protocols used on human participants in the literature and to discuss their applicability.

2.2 Question in Detail

Each question will now be outlined in more detail

1. This question is looking for potential bias in the sample populations and is not related to sample size. A clear and non-biased enrolment is considered to be one in which the enrolled participant details (e.g. age, gender, diagnosis) are outlined and the method of enrolment is such that the risk of bias is reduced, i.e. the group were randomly selected, form a consecutive group of patients, details of where they were enrolled from is given etc.
2. This question pertains to a comparison group, from which the generalisability of the technique/outcomes may be better understood. What constitutes a matched cohort is up to your interpretation.
3. Question 3 relates to the imaging methods and asks whether or not they are sufficiently clear so that a reader may be reasonably able to replicate their method or similar. If the imaging methods vary throughout the study, without reasonable justification, then the method is less interpretable/repeatable and hence a point should be docked from the maximum mark of 2. A mark should only be docked if it is clear that the method changed, without good cause, during the study.
4. As above, is the injection protocol, if used, reproducible to the reader.
5. Here we are assessing if the way the data is to be interpreted/produced is clear. This includes post processing, scoring images, making a diagnosis, measuring signals etc. A mark is only to be given if the method is clear and the reporting of the result is clear.
6. Participant compliance is a clear test of the utility of the imaging method; if the compliance is low the applicability is low. High compliance is considered to be > 80% of the enrolled participants completing the imaging. This question is out of 1 for non-contrast enhanced studies, but an additional 1 point is available to contrast enhanced studies which document potential/actual side effects of the injection.
7. When the methods include measurement of some value, are the methods of measurement and any subsequent numerical and statistical analysis clear to interpret.
8. Are the actual values (or mean, range etc.) actually displayed in the article and are they clear? Ideally this will also include some indication of the variability of the data, but it may be subjective as to whether this is necessary.
9. In order for a reader to be confident that the method outlined is worth attempting, the potential practical and imaging considerations and potential cofounders should be discussed. This may include: length of scan, patient comfort, motion blurring induced by breathing and vascular motion, vascular signal contamination, partial volume effects etc. Ideally a method of reducing the influence of these issues should also be described and implemented. Only 1 mark is to be awarded for studies referring to potential issues (regardless of whether they actually observed it to be problematic) and a second can be awarded if they attempt to control these confounders.

3. Result

Prior to scoring, Reviewer 1 went through all shortlisted papers and removed those which lost ≥ 6 marks due to too many N/A marks. As an example, if a paper reviewing local practice did not detail participant selection, Q1 and Q2 may score N/A. If all other questions could be marked this paper was not removed at this stage. If, however they also did not detail the imaging protocol, then Q1-3 are all scored N/A meaning 6 points could not be awarded and hence the paper was excluded. This cut-off was considered sufficient to remove articles lacking sufficient relevant information without introducing a severe bias against non-CE studies; for which 2 points were not applicable by default (see Supplementary Table 1). This initial screen led to 14 papers being excluded on these grounds.

Next, each reviewer individually assessed the remaining papers, awarding marks using the schema in Supplementary Table 1. Articles scoring ≥ 60% of the available marks by both reviewers were considered of good quality and with low risk of bias, and were immediately selected for inclusion in the review. Where there was disagreement regarding inclusion of an article this was discussed between reviewers and agreed by consensus. If there was still disagreement a third reviewer with over 15 years of MRI experience made the final decision.

4. References

[1] National Heart Lung and Blood Institute, Quality Assessment Tool for Case Series Studies, Study Qual. Assess. Tools. (2020). https://www.nhlbi.nih.gov/health-topics/study-quality-assessment-tools (accessed January 5, 2020).

[2] S.H. Downs, N. Black, The feasibility of creating a checklist for the assessment of the methodological quality both of randomised and non-randomised studies of health care interventions, J. Epidemiol. Community Health. 52 (1998) 377–384. https://doi.org/10.1136/jech.52.6.377.
